# Analysis of Antioxidant Capacity and Antimicrobial Properties of Selected Polish Grape Vinegars Obtained by Spontaneous Fermentation

**DOI:** 10.3390/molecules26164727

**Published:** 2021-08-04

**Authors:** Justyna Antoniewicz, Karolina Jakubczyk, Paweł Kwiatkowski, Dominika Maciejewska-Markiewicz, Joanna Kochman, Ewa Rębacz-Maron, Katarzyna Janda-Milczarek

**Affiliations:** 1 Department of Human Nutrition and Metabolomics, Pomeranian Medical University in Szczecin, 24 Broniewskiego Street, 71-460 Szczecin, Poland; kaldunskajustyna@gmail.com (J.A.); jakubczyk.kar@gmail.com (K.J.); dmaciejewska@pum.edu.pl (D.M.-M.); kochmaan@gmail.com (J.K.); 2 Department of Diagnostic Immunology, Chair of Microbiology, Immunology and Laboratory Medicine, Pomeranian Medical University in Szczecin, 72 Powstańców Wlkp. Street, 70-111 Szczecin, Poland; pawel.kwiatkowski@pum.edu.pl; 3 Department of Ecology and Anthropology, Institute of Biology, University of Szczecin, 13 Wąska Street, 71-415 Szczecin, Poland; ewa.rebacz-maron@usz.edu.pl

**Keywords:** polyphenol content, *Escherichia coli*, *Staphylococcus aureus*, *Candidia albicans*, chaptalisation process, spontaneous fermentation

## Abstract

Nowadays, products of natural origin with health-promoting properties are increasingly more common. Research shows that fruit vinegars can be a source of compounds with antioxidant activity. Research on the total antioxidant capacity, total phenolic content, and antimicrobial properties against *Staphylococcus aureus*, *Escherichia coli*, and *Candida albicans* of grape vinegars were conducted. Moreover, gas chromatography was used to measure acetic acid content in the vinegars. The research material consisted of vinegars produced from five different grape varieties. For each variety, two variants were prepared: with and without the addition of sugar in the fermentation process. The highest antimicrobial activity against all micro-organisms was observed in vinegar produced from Solaris grapes with added sugar. The highest polyphenol content was observed in vinegar produced from the Prior grape variety with added sugar and the highest total antioxidant capacity is the Johanniter grape variety with added sugar. The vinegars examined in this study differed, depending on grape variety, in terms of antimicrobial properties, antioxidant capacity, total phenolic content, as well as acetic acid content. Sugar addition caused significant differences in the antioxidant capacity of vinegar samples.

## 1. Introduction

Vinegar is a product of a two-staged fermentation process [1,2]. Firstly, fermentable sugars are converted to ethanol by the yeasts in anaerobic conditions. Subsequently, ethanol is transformed into acetic acid by bacteria of the *Acetobacter* genus during the oxidation process, also known as acetic acid bacteria (AAB) in aerobic conditions [3]. The most common substrates used for vinegar production are products with a high sugar content [4,5], but they can also be produced from alcohols, including wine [6]. Acetic acid is the main product of the fermentation process, but small amounts of tartaric acid and citric acid are also present [7]. Apart from organic acids, vinegar also contains colouring matter, mineral salts, and other fermentation products, such as esters, ketones, and aldehydes, which are responsible for vinegar’s distinctive flavour and aroma [8,9]. The traditional vinegar production process takes about 30 days. It is conducted by AAB, which are widespread in the environment, occurring particularly in food material containing saccharides [10].

Fruit vinegars, including wine vinegars, contain many compounds with antioxidant properties, which may originate from the source material (i.e., fruit); however, their content may change during the acetic fermentation process, e.g., by increasing total flavonoid and total phenolic content during fermentation [11]. The phenolic compounds found in vinegar not only increase its antioxidant capacity but also affect its colour and astringency. The quality of vinegar and its chemical and sensory properties may also be determined by oxygen availability and composition of the bacteria starter cultures. It should be emphasized that the vinegar fermentation process is also an aerobic reaction, and oxygen is an essential factor for the growth of bacteria participating in this process [12].

The consumption of vinegar is associated with several health benefits [13,14,15,16]. Vinegars are also known for their strong antimicrobial properties [17]. These properties are mainly due to the content of acetic acid, which inhibits the development of pathogenic and food spoilage organisms [18]. Bacteria such as *Escherichia coli*, *Staphylococcus aureus* and yeasts like *Candida albicans* are part of normal human microbiota [19]. However, pathogenic strains of these micro-organisms may contribute to disorders of the gastrointestinal tract [20], skin and soft tissues [21], circulatory system, respiratory tract [22], and the urogenital system [23]. Identification of non-antibiotic antimicrobial agents may help reduce the use of antibiotics in the treatment of certain conditions and prevent further development of antibiotic resistance [24].

Spontaneous fermentation is a vinegar production method in which the naturally occurring microbiota of the plant material is involved. The composition of the grape microbiome is comprehensive, consisting of a high range of yeasts, bacteria, and filamentous fungi [25]. These micro-organisms play a significant role in the fermentation process, metabolising the sugars in grapes and influencing the production of a whole set of secondary metabolites that affect the final quality of the fermented product [26]. Sugar addition before fermentation is a common practice in the domestic production of alcoholic beverages and other fermented products. The study aimed to determine the antioxidant capacity, total phenolic content, and antimicrobial properties against *E. coli, S. aureus*, and *C. albicans* of grape vinegars produced from different varieties of the common grape vine (*Vitis vinifera* L.) by spontaneous fermentation.

## 2. Materials and Methods

### 2.1. Grape Vinegars

The vinegars used in the study were produced from the fruit of wine grape varieties (*Vitis vinifera* L.) obtained from a vineyard in the West Pomerania (Zachodniopomorskie) region of Poland (53°15′35″ N 14°43′24″ E) in September 2018. The study used white grape varieties: Solaris, Johanniter, and Souvignier gris, as well as red varieties, including Prior and Cabernet cortis. Grapes were collected from individual plants of each variety during the full ripening stage of the grape. For each variety, vinegars were prepared according to two different procedures. In the first variant, only crushed fruit and distilled water were used at a 1:1 mass ratio. In the second variant, the chaptalisation process was also used: a solution of distilled water and table sugar (70 g sugar per 1 L of water) was added to the fruit (also 1:1 mass ratio). Whole grapes were used to prepare the vinegar, with the skins and seeds remaining (the stalks were removed). Vinegar was produced by spontaneous fermentation at a temperature of 24 °C over two months, conducted by the natural flora inhabiting the fruit. Both variants of the fermentation process were performed in triplicate.

### 2.2. Determination of Total Antioxidant Activity

The antioxidant activity of samples was measured by spectrophotometry (Agilent 8453 UV-visible spectrophotometer) using a synthetic radical, DPPH (2,2-diphenyl-1-picrylhydrazyl, Sigma–Aldrich, Darmstadt, Germany). Trolox (6-hydroxy-2,5,7,8-tetramethylchroman-2-carboxylic acid, Sigma–Aldrich, Darmstadt, Germany) was used as the antioxidant standard. The antioxidant activity of samples was measured according to Brand–Williams et al. and Pekkarinen et al. [27,28]. The spectral absorbance was measured immediately at 518 nm. All assays were performed in triplicate. In order to determine the Trolox Equivalence Antioxidant Capacity (TEAC), a Trolox calibration curve was constructed, using dilutions of the stock solution with a concentration of 2.5 mmol/dm^3^. Quantitative determinations were performed using the calibration curve method (r^2^ = 0.913) by plotting the absorbance against nine concentrations (in the range 0.02 ÷ 2.0 mmol/dm^3^) of Trolox ethanol solutions as described by Śnieżek et al. [29]. The results were expressed as mg Trolox Equivalent/L of liquid.

### 2.3. Determination of Total Phenolic Content

Polyphenol content was assessed using the Folin–Ciocalteu reagent [30]. The absorbance was measured at 765 nm (Agilent 8453 UV). Polyphenol content was calculated from the calibration curve plotted using gallic acid as the reference standard. The results are shown as mg of gallic acid in 1 L of liquid (mg GAE/1 L). All assays were performed in triplicate.

### 2.4. Bacterial Strains and Culture Conditions

The following reference strains were used in the study: *Staphylococcus aureus* ATCC 29213, *Escherichia coli* ATCC 25922 and *Candida albicans* ATCC 10231. Bacteria were inoculated on Columbia agar supplemented with 5% sheep blood (bioMeriéux, Warsaw, Poland) and then incubated at 37 °C for 24 h under aerobic conditions. Yeasts were cultured on Sabouraud agar (bioMeriéux, Warsaw, Poland) and incubated at 37 °C for 48 h under aerobic conditions.

### 2.5. Determination of the Antimicrobial Activity of Grape Vinegars

Antimicrobial activity defined by minimum inhibitory concentration (MIC) of vinegars against bacteria was determined using the microdilution method in Mueller–Hinton broth (MHB, Sigma–Aldrich, Darmstadt, Germany) in accordance with the guidelines of the Clinical and Laboratory Standards Institute (CLSI) [31]. The MIC of vinegars against yeasts was determined using the microdilution method recommended by the CLSI in Sabouraud broth (SAB, Sigma–Aldrich, Darmstadt, Germany) [32]. The following dilutions were prepared for all vinegars: 500 µL/mL–3.9 µL/mL. Samples containing 50 µL of the respective vinegar concentrations were placed in 96-well microtiter plates. Afterwards, 50 µL of a bacterial suspension at 10^6^ CFU/mL or 50 µL of yeast suspension at 1 × 10^3^–5 × 10^3^ CFU/mL were added into each well of the plate. After incubation for 24 h at 37 °C, the MIC for the respective vinegars was determined by adding 20 µL of 0.02% resazurin (Sigma–Aldrich, Darmstadt, Germany) into the wells [33]. A change of colour from blue to pink after a 3-h incubation with resazurin at 37 °C indicated the presence of bacteria/yeast. The first well to remain blue determined the MIC value. Additionally, the assay included a positive growth control (MHB + bacterial suspension/SAB + yeast suspension) and a negative sterility control (MHB/SAB). For each vinegar, the assay was conducted in duplicate.

### 2.6. Determination of the Acetic Acid Content

Chromatographic analyses were conducted using the Agilent Technologies 1260 A GC system with a flame ionization detector (FID). A fused-silica capillary column with a free fatty acid phase (DB-FFAP, 30 m × 0.53 mm × 0.5 um) was used. The carrier gas was hydrogen at a flow rate equal to 14.4 mL/min. The initial temperature (100 °C) was maintained for 0.5 min, then raised to 180 °C with ramping of 8 °C/min to be constant for 1 min. Subsequently, the temperature was increased to 200 °C (ramping 20 °C/min) to eventually reach 200 °C and be sustained for 5 min. The injection volume was 5 uL, and the run time of a single analysis was 17.5 min. Results were presented as a percentage of acids content, according to the surface area. Moreover, the amount of acetic acid was evaluated using the calibration curve method (mM of acetic acid/L). 

### 2.7. Determination of pH

The pH of the samples was determined by a pH meter (SCHOTT Instruments; SI Analytics Mainz, Mainz, Germany).

### 2.8. Statistical Analysis

All of the samples (prepared in three repetitions) were analysed in all the experiments, and all the assays were conducted at least in triplicate. The statistical analysis was performed using Stat Soft Statistica 13.0 and Microsoft Excel 2017. The results are expressed as mean values and standard deviation (SD). Distributions of values for each parameter were analysed using the Shapiro–Wilk test. In order to assess the differences between examined parameters, the Kruskal–Wallis test was used. Differences were considered significant at *p* ≤ 0.05.

## 3. Results

Vinegars were analysed by gas chromatography (GC) to determine acetic acid content. The results, presented in Table 1, indicate that the acetic acid content in the tested samples ranged from 90.12 to 469.94 mM/L. Acetic acid was also the dominant organic acid found in the analysed vinegars (96.41–98.87% of the total organic acid content). Other acids found in the analysis were: propionic acid, butyric acid, and pentanoic acid; however, their total amount did not exceed 4%. The highest acetic acid content was observed in the vinegar sample of the Solaris variety (variant with added sugar). The vinegar variants subjected to the chaptalisation process contained higher levels of acetic acid than their counterparts without added sugar. The exception was the vinegar of the Cabernet cortis variety, where the variant without added sugar contained a higher content of acetic acid compared with the variant with added sugar. However, these differences were not statistically significant. A statistically significant difference was observed only between the variants of Solaris vinegars (*p* = 0.032359). In most cases, significant differences were observed in the acetic acid content between vinegars prepared from different grape varieties (Table 1).

Table 2 presents MIC values of the analysed vinegars against *S. aureus*, *E. coli,* and *C. albicans*. The highest antimicrobial activity against all micro-organisms included in the analysis was observed in the vinegar produced from Solaris grapes with added sugar (MIC = 62.5 µL/mL against bacteria, MIC = 125 µL/mL against yeast). Likewise, the vinegar produced from Solaris grapes by fermentation without added sugar demonstrated strong antimicrobial activity against *S. aureus* and *E. coli* (MIC = 62.5 µL/mL). Most vinegar samples showed a relatively low capacity for inhibiting the growth of *C. albicans*. The lowest antimicrobial activity was observed in the vinegar produced from Johanniter grapes without added sugar (MIC > 500 µL/mL). In the majority of analysed samples, there was a clear difference in the antimicrobial activity of vinegars obtained with and without the addition of sugar.

The total antioxidant capacity of vinegars expressed as mg of Trolox Equivalent per 1 L of vinegar ranged from 89.04 ± 21.25 mg TE/L to 203.69 ± 35.73 mg TE/L (Table 3). The highest antioxidant capacity was found in vinegar produced from Johanniter grapes with added sugar. High antioxidant potential was also obtained by both variants of vinegar produced from Solaris grapes (185.76 ± 14.78 mg TE/L in the variant with no added sugar and 180.76 ± 58.62 mg TE/L in the variant with added sugar) and in the vinegar produced from Cabernet cortis grape variety with added sugar (177.41 ± 25.43 mg TE/L). The lowest antioxidant capacity was observed in vinegar produced from Souvignier gris grapes without sugar (89.04 ± 21.25 mg TE/L). Statistical analysis revealed statistically significant differences for all vinegars included in the study, between the variants produced with the addition of sugar in the fermentation process, and those produced without it. In the case of vinegars produced from Johanniter, Cabernet cortis, Souvignier gris, and Prior grape varieties, the variants produced with added sugar showed a statistically significant, higher antioxidant capacity compared with their counterparts without added sugar (*p* = 0.00176, *p* = 0.00041, *p* = 0.000166, and *p* = 0.00376, respectively).

The total phenolic content in the analysed vinegars varied (Table 3). The highest values were observed in vinegar produced from Prior grapes with added sugar (1437.77 ± 14.74 mg GAE/L), whereas the lowest was in vinegar produced from Solaris grapes without added sugar (289.8 ± 38.04 mg GAE/L). Both variants of vinegar produced from the Prior grape variety had a statistically significant, higher polyphenols content compared with other samples included in the analysis. Moreover, in the case of vinegars produced from the Solaris, Souvignier gris, and Prior grape varieties, a higher phenolic content was observed in the vinegar produced with added sugar than in the variant produced without added sugar. The differences were statistically significant (respectively, *p* = 0.00013, *p* = 0.00013 and *p* = 0.00016). 

A pH analysis was also performed (Table 3). The highest pH value was observed for Johanniter vinegar without added sugar (3.517 ± 0.038) and the lowest for grape vinegar of the Souvignier gris variety with added sugar (2.955 ± 0.005). In most cases, statistically significant differences were observed.

## 4. Discussion

In recent years, there has been a growing interest in the antimicrobial and health-promoting properties of substances and products of plant origin. Modern lifestyles and environmental pollution can be linked to increased free-radical reactions, which may be why nourishment with a high antioxidant content are attracting special attention. Vinegar may be regarded as one of these products.

There is a range of factors, including ambient temperature, sugar content in the source material or the availability of oxygen, which may affect the speed of the acetic fermentation process and the characteristics of the final product [34]. According to Valles et al., the quality and chemical composition of the final product is also affected by the strain of yeast involved in fermentation [35].

In our study, the flora naturally occurring on grapes was involved in the spontaneous fermentation process. It is the oldest known method of vinegar production. However, this process takes longer compared with current production methods [36]. Currently, a gradual return of interest to traditional fermented food production methods can be observed [37]. However, differentiating the fruit flora that took part in the fermentation may have influenced the differentiation of the obtained results. The residual microflora of grapevines is highly diversified, and its composition is determined by many factors, such as the geographic location of the cultivation place, rainfall intensity, temperature, and grape variety [38].

Unfortunately, there are currently only a few reports on vinegars obtained by the spontaneous fermentation method. Ubeda et al. [39] observed that vinegar obtained by spontaneous fermentation of persimmon fruit showed a higher antioxidant potential compared with vinegar obtained from the inoculation method. However, these differences were not statistically significant.

In the present study, it was demonstrated that grapevine variety, as well as the addition of sugar in the fermentation process, affected the quality of the finished vinegar. In the analysis of antioxidant capacity, it was observed that vinegars obtained with added sugar showed greater potential for inhibiting free-radical reactions than their counterparts produced without added sugar. This regularity was not observed only in the case of vinegars produced from the fruit of Solaris variety. A similar relationship was observed in the analysis of total polyphenols content. In the case of vinegars from the Solaris, Souvignier gris, and Prior grape varieties, the variants prepared with added sugar showed statistically significant, higher TPC content. The addition of sugar increased the amount of substrate for the yeasts, which may have affected the intensity of their activity. During fermentation, some changes in the composition of the polyphenols occur since they are involved in various reactions such as co-pigmentation, cycloaddition, polymerization, and oxidation [40]. However, in some cases, increased osmotic pressure may have a restrictive effect on the development of certain yeast strains [41].

The study also demonstrated high antioxidant capacity, expressed as Trolox Equivalent, reaching 203.69 mg TE/L for the vinegar produced with Johanniter grapes. Similar findings were obtained by Budak et al., who examined the antioxidant capacity of grape vinegars and observed results ranging from 28 to 132 mg TE/L of vinegar [42]. Our findings were significantly higher than those observed by Sengun et al. [43], where the antioxidant capacity of grape vinegar amounted to 0.019 mg TE/L. However, it should be mentioned that in our study, we used grapes together with skins and seeds for vinegar preparation. It is known that seeds and grape skins contain a large part of the total polyphenol content for the grape (mainly flavan-3-ols in seeds and anthocyanins and stilbenes in skins) [1]. In the study by Sengun et al., the examined vinegars were bought from local markets and their exact preparation method was not provided. On the other hand, Kadiroğlu noted significantly higher antioxidant capacity in grape vinegars compared with our study, with values up to 420 mg TE/L [44], where the exact preparation of the examined vinegars was also not provided. The antimicrobial properties, as well as the antioxidant activity of grape vinegars, may be attributed to the presence of both polyphenolic compounds and organic acids [45] where apart from acetic acid, there are also others including citric acid, tartaric acid [46], gallic acid, chlorogenic acid, caffeic acid, syringic acid, and ferulic acid [47]. Most of the organic acids in vinegars arise during the fermentation process; a small amount also comes from the raw material [47].

Acetic acid was the main organic acid found in the examined vinegars and was responsible for powerful antimicrobial effects. Its concentration in the analysed samples ranged from 90.12 to 469.94 mM/L. An analysis of commercial vinegars by Kong et al. [48] showed that the acetic acid content ranged from 58.96% to 91.36% of the total organic acid content. In our study, the content of this acid in the total amount of organic acids was not under 96.41%. Acetic acid has antimicrobial activity [49]. An analysis of organic acids’ influence on foodborne pathogenic bacteria showed that acetic acid had the strongest inhibitory effect on the growth of *Escherichia coli* O157: H7, compared to lactic, citric, and malic acid [50]. The addition of vinegar also inhibited the growth of E. coli in the refrigerator-stored food [51]. A 10% vinegar solution was effective in inhibiting the development of *Candidia* spp. [52]. A study on the antimicrobial properties of apple cider vinegar against *E. coli*, *C. albicans,* and *S. aureus* showed that the type of micro-organism determined the dilution of vinegar at which their inhibited development was observed [53]. It has been suggested that acetic acid affects bacterial metabolism, e.g., inhibiting enzymatic activities, perturbation of membrane function, impairing nutrient transport, and weakening metabolic activity [54,55]. In our study, the most powerful antimicrobial activity and the highest acetic acid content were demonstrated for the vinegar produced from the Solaris grape variety with added sugar. High acetic acid content and strong antimicrobial properties were also observed for the variant without added sugar of the Solaris variety. Simultaneously, vinegar from the Johanniter variety (variant without sugar) had the lowest acetic acid content and showed the weakest antimicrobial properties against the tested micro-organisms. It was also observed that vinegars subjected to the chaptalisation process contained a higher acetic acid content than their counterparts without added sugar.

In our study, we observed differences in antimicrobial properties between samples with and without added sugar. The process of adding sugar to grape is called chaptalisation. In the first step of acetic acid fermentation, yeast converts glucose to ethanol. The purpose of chaptalisation is to provide more substrate for yeast, resulting in a higher ethanol content [56]. This alcohol is produced in the first stage of fermentation and has proven antimicrobial properties [57]. However, the further acetic acid fermentation process significantly lowers ethanol content. It was observed that vinegars subjected to the chaptalisation process, in most cases, contained a higher acetic acid content than their counterparts without added sugar. However, in most cases, these differences were not statistically significant.

Different conditions of vinegar preparation (sugar addition) influenced their antimicrobial properties. In most cases, variants of vinegars with added sugar showed higher antimicrobial properties, which were mainly associated with a higher acetic acid content. However, the properties investigated in this study may also be associated with compounds contained in the source material [58]—fruit—a rich source of polyphenols, which are most abundant under the fruit skin and in the seeds [59]. Antioxidants are also found in other parts of the plant, including the skin and stalks [15]. In our study, whole grapes, including seeds and skins, were used to prepare the vinegar samples. Polyphenolic compounds are produced in fruit during plant development in response to stress factors [60]. Polyphenols include anthocyanins, flavanols, flavonols, stilbenes, and phenolic acids [61].

In grapes, flavanols are present in the form of catechins, epicatechin, and proanthocyanidins. They account for 13–30% of the total phenolic content [59,62]. Flavonols are the second most abundant flavonoids in grapes and are found only in grape skin. The amount of individual compounds from this category depend on the variety of grapevine [62,63,64]. Anthocyanins are the pigment in grape skin, and their presence is characteristic of red grape varieties. White grape varieties contain considerable amounts of flavan-3-ols [62,65].

The total phenolic content in the analysed samples varied markedly, ranging from 289.8 mg GAE/L to 1437.77 mg GAE/L. The highest content of these compounds was found in vinegars produced from the fruit of the Prior variety. This significant advantage from the content of the polyphenolic compounds may be caused by the dark colour of this grape variety. Some research indicates that dark-coloured plants contain more polyphenols than their pale varieties [66,67]. They also indicated that the skin of the fruit contributes to the difference in phenol content. The total phenol content in red skin is higher because the skin of the white fruit cannot produce anthocyanins [68].

The analysis of total phenolic content by Kadiroğlu revealed a similar TPC range for grape-based vinegars at 109.66–919.95 mg GAE/L [44]. TPC in fruit vinegars observed by Sengun et al. [43] ranged between 933 mg GAE/L and 1162 mg GAE/L. In the aforementioned study, the highest total phenolic content was found in blackberry vinegar, whereas grape vinegar had 1025 mg GAE/L. In the study conducted by Bakir et al., the TPC in grape vinegars was lower than in the present study, ranging between 260 mg GAE/L and 680 mg GAE/L [42]. A similar report on the antioxidant and antimicrobial characteristics of vinegars was presented by Ozturk et al. [9]. They analysed 25 samples of traditional homemade and industrial vinegars and demonstrated that homemade grape vinegars had the highest TPC content and antioxidant capacity. Apart from the choice of raw material, polyphenol contents are also influenced by the vinegar production process, but mainly the maturation process. However, Bakir et al. [11] did not observe statistically significant changes in TPC between the grape wine and vinegar obtained from it. On the other hand, a study comparing wines from persimmon fruit obtained from spontaneous and inoculated fermentation showed that spontaneously fermented wine had a higher content of flavonoids, phenols, a lower content of alcohol, and volatile compounds [69]. In spontaneous fermentation, non-*Saccharomyces* yeasts, also known as wild yeasts, dominated the process [70]. Research shows that yeasts act in two ways during the fermentation process: They can capture polyphenols and release antioxidant compounds other than polyphenols from inside the cells and from the cell wall [39], i.e., by the release of bound and conjugated phenolic acids to their free form after cell wall degradation [71]. It was observed that the strain of yeast involved in alcoholic fermentation affected the profile of organic acids [72] as well as TCP and antioxidant potential [73].

When analysing the TPC and TEAC of our samples, it can be observed that the value of both parameters is not proportional. These differences may be due to different characteristics between the two methods. In order to analyse the total antioxidant potential, expressed as Trolox Equivalent, we used the DPPH free radical, which reacts with polyphenols (catechins, proanthocyanidins) but not with phenolic acids and sugars [74]. Analysing the antioxidant potential is a complex process because plants contain two main types of antioxidants: polar (such as phenolics) and non-polar; there is no single method suitable for assessing both types. Moreover, the complex composition of plant extracts can lead to contradictory results if the antioxidant activity is evaluated by a single method [75]. Therefore, further research is needed.

Literature data suggest that polyphenols, depending on their structure and concentration, may affect bacterial growth and metabolism [76,77]. Rodríguez-Vaquero et al. [78] demonstrated the inhibitory effect of several wine varieties on the growth of *Escherichia coli* strains, which is directly proportional to the polyphenol content in the wines included in the analysis. A similar regularity was observed in the present study, where vinegars produced from Prior grapes, which had the highest TPC, also displayed powerful inhibitory effects against *E. coli*.

Papadopoulou et al. [79] reported antimicrobial activity of red wine extracts against pathogenic strains of *S. aureus*, *E. coli*, and *C. albicans.* The extracts used in their study were alcohol-free, indicating that their activity may have been due to the presence of bioactive compounds, including polyphenols. The authors also suggested that the analysed pathogenic micro-organisms exhibited different degrees of sensitivity to the phenolic concentration in the extracts. The tested extracts were most effective against *S. aureus* and less effective against *E. coli. C. albicans* showed the highest resistance to the substances used [66]. In the present study, the minimum inhibitory concentration of tested vinegars against *C. albicans* was markedly higher than *S. aureus* and *E. coli.* Kadiroğlu’s study with a variety of fruit-based vinegars showed that grape vinegars presented powerful biocidal activity against *E. coli* and *P. aeruginosa*. A stronger effect was observed only in the case of balsamic vinegars [44].

A strong inhibitory effect on the growth of *S. aureus* and *E. coli* was also noted in defatted grape seed extracts [80]. Notably, the variability of antimicrobial properties reflects the differences of phenolic content in different morphological parts of *Vitis vinifera* L. The antibacterial activity of fermented pomace was significantly higher than whole fruit extracts from grapes [81].

## 5. Conclusions

In conclusion, the grape vinegars included in the study differed in terms of antimicrobial activity, antioxidant capacity, and total phenolic content. These differences were strongly associated with the grape variety used in vinegar preparation. Significant differences were also observed in the analysis of antioxidant capacity between samples prepared with and without added sugar in the fermentation process. The substantial variability of the total phenolic content in the analysed samples may be associated with different properties of the raw material, i.e., different varieties of *V. vinifera* L. fruits. This study proves that grape vinegars obtained by spontaneous fermentation may be a potential source of compounds with antioxidant and antimicrobial effects. Furthermore, adding sugar before the fermentation process, which is a common practice in homemade fermented products, appears to be the best method for reaching a higher content of compounds with antioxidant activity and acetic acid content.

## Figures and Tables

**Table 1:** Acetic acid content in the analysed vinegar samples.

Vinegar Sample	Acetic Acid [mg/L]	Acetic Acid in Total Organic Acid Amount [%]
Johanniter	90.12 ± 23.24 ^3,4,7,8,9,10^	96.41 ± 3.59
Johanniter + sugar	154.26 ± 77.92 ^3,4,8^	98.45 ± 0.24
Cabernet cortis	184.53 ± 26.49 ^1,2,4,5,6^	98.53 ± 0.03
Cabernet cortis + sugar	119.56 ± 58.00 ^1,2,3,5,6,7,9,10^	97.53 ± 0.06
Solaris	448.48 ± 22.84 ^3,4,8^	98.13 ± 0.79
Solaris + sugar	469.94 ± 43.37 ^3,4,7,8,10^	98.97 ± 0.56
Souvignier gris	237.94 ± 21.90 ^1,4,6^	98.74 ± 0.34
Souvignier gris + sugar	277.54 ± 48.09 ^1,2,5,6^	98.50 ± 0.70
Prior	276.02 ± 7.38 ^1,4^	97.74 ± 0.04
Prior + sugar	342.14 ± 99.99 ^1,4,6^	98.87 ± 0.72

Data represent the mean values ± standard deviations of the three biological × three technical replicates. Different numbers (1–10) represent different samples of the vinegar. ^1^ Johanniter, ^2^ Johanniter + sugar, ^3^ Cabernet cortis, ^4^ Cabernet cortis + sugar, ^5^ Solaris, ^6^ Solaris + sugar, ^7^ Souvignier gris, ^8^ Souvignier gris + sugar, ^9^ Prior, ^10^ Prior + sugar. Numbers in the superscript assigned to the presented value represent statistically significant differences (*p* ≤ 0.05).

**Table 2:** Minimum inhibitory concentration (µL/mL) of vinegar samples against *Staphylococcus aureus*, *Escherichia coli*, and *Candida albicans*.

Vinegar Sample	Minimum Inhibitory Concentration (µL/mL)		
	*Staphylococcus aureus*	*Escherichia coli*	*Candida albicans*
	ATCC 29213	ATCC 25922	ATCC 10231
Johanniter	>500	>500	>500
Johanniter + sugar	125.0 ± 0.0	125.0 ± 0.0	500.0 ± 0.0
Cabernet cortis	500.0 ± 0.0	125.0 ± 0.0	500.0 ± 0.0
Cabernet cortis + sugar	250.0 ± 0.0	125.0 ± 0.0	500.0 ± 0.0
Solaris	62.5 ± 0.0	62.5 ± 0.0	250.0 ± 0.0
Solaris + sugar	62.5 ± 0.0	62.5 ± 0.0	125.0 ± 0.0
Souvignier gris	187.5 ± 88.4	125.0 ± 0.0	187.5 ± 88.4
Souvignier gris + sugar	125.0 ± 0.0	62.5 ± 0.0	250.0 ± 0.0
Prior	125.0 ± 0.0	62.5 ± 0.0	250.0 ± 0.0
Prior + sugar	125.0 ± 0.0	62.5 ± 0.0	250.0 ± 0.0

**Table 3:** Trolox equivalence antioxidant capacity (TEAC), total polyphenol content (TPC) and pH value in analysed vinegar samples.

Vinegar Sample	TEAC	TPC	pH
Johanniter	129.51 ± 21.98 ^2,5,6,7,8,9^	334.6 ± 4.7 ^6,8,9,10^	3.517 ± 0.038 ^1,2,3,4,5,6,7,8,9,10^
Johanniter + sugar	203.69 ± 35.73 ^1,3,7,8,9,10^	331.1 ± 8.8 ^6,8,9,10^	3.225 ± 0.093 ^1,3,6,8,9^
Cabernet cortis	135.24 ± 22.2 ^2,4,5,6,7^	328.4 ± 6.1 ^6,8,9,10^	3.325 ± 0.010 ^1,2,4,5,6,7,8,10^
Cabernet cortis + sugar	177.41 ± 25.43 ^3,6,7,8,9^	325.7 ± 10.4 ^6,8,9,10^	3.215 ± 0.075 ^1,3,6,8,9^
Solaris	185.76 ± 14.78 ^1,3,7,8,9,10^	289.8 ± 38.0 ^6,8,9,10^	3.205 ± 0.150 ^1,3,6,8,9^
Solaris + sugar	180.76 ± 58.62 ^1,3,4,7,8,9,10^	724.7 ± 47.0 ^1,2,3,4,5,7,8,9,10^	3.045 ± 0.028 ^1,2,3,4,5,6,7,8,9,10^
Souvignier gris	89.04 ± 21.25 ^1,2,3,4,5,6,10^	308.1 ± 14.4 ^6,8,9,10^	3.180 ± 0.014 ^1,3,6,8,9^
Souvignier gris + sugar	114.8 ± 17.31 ^1,2,4,5,6,10^	638.2 ± 14.2 1^,2,3,4,5,6,7,9,10^	2.955 ± 0.005 ^1,2,3,4,5,6,7,8,9,10^
Prior	107.42 ± 3.04 ^1,2,4,5,6,10^	1310.9 ± 43.2 ^1,2,3,4,5,6,7,8,10^	3.340 ± 0.029 ^1,2,4,5,6,7,8,9,10^
Prior + sugar	174.0 ± 4.2 ^2,5,6,7,8,9^	1437.8 ± 14.7 ^1,2,3,4,5,6,7,8,9^	3.235 ± 0.047 ^1,3,6,8,9^

Data represent the mean values ± standard deviations of the three biological × three technical replicates. Different numbers (1–10) represent different samples of the vinegar: ^1^ Johanniter, ^2^ Johanniter + sugar, ^3^ Cabernet cortis, ^4^ Cabernet cortis + sugar, ^5^ Solaris, ^6^ Solaris + sugar, ^7^ Souvignier gris, ^8^ Souvignier gris + sugar, ^9^ Prior, ^10^ Prior + sugar. Numbers in the superscript assigned to the presented value represent statistically significant differences (*p* ≤ 0.05). Total antioxidant capacity is expressed as mg of TE (Trolox Equivalent) per 1 L of vinegar. Total polyphenols content is expressed as mg gallic acid per 1 L of vinegar.

## References

[1] Solieri L., Giudici P., Solieri L., Giudici P. (2009). Vinegars of the world. Vinegars of the World.

[2] Bekatorou A. (2019). Advances in Vinegar Production.

[3] Li S., Li P., Feng F., Luo L.X. (2015). Microbial diversity and their roles in the vinegar fermentation process. Appl. Microbiol. Biotechnol..

[4] Bamforth W. (2005). Vinegar. Food, Fermentation and Micro-Organisms.

[5] Hutchinson U.F., Jolly N.P., Chidi B.S., Ngongang M.M., Ntwampe S.K.O. (2019). Vinegar Engineering: A Bioprocess Perspective. Food Eng. Rev..

[6] Tesfaye W., Morales M.L., García-Parrilla M.C., Troncoso A.M. (2002). Wine vinegar: Technology, authenticity and quality evaluation. Trends Food Sci. Technol..

[7] Hailu S., Admassu S., Jha K. (2012). Vinegar Production Technology—An Overview. Beverage Food World.

[8] Vikas Bhat S., Akhtar R., Amin T. (2014). An Overview on the Biological Production of Vinegar. Int. J. Fermented Foods.

[9] Ozturk I., Caliskan O., Tornuk F., Ozcan N., Yalcin H., Baslar M., Sagdic O. (2015). Antioxidant, antimicrobial, mineral, volatile, physicochemical and microbiological characteristics of traditional home-made Turkish vinegars. LWT Food Sci. Technol..

[10] Hemke J., Rasane P., Kaur S., Kumbhar P., Singh J. (2019). Vinegar: A traditional functional food. Think India J..

[11] Bakir S., Toydemir G., Boyacioglu D., Beekwilder J., Capanoglu E. (2016). Fruit antioxidants during vinegar processing: Changes in content and in vitro bio-accessibility. Int. J. Mol. Sci..

[12] Mas A., Torija M.J., García-Parrilla M.D.C., Troncoso A.M. (2014). Acetic acid bacteria and the production and quality of wine vinegar. Sci. World J..

[13] Ashchyan H., Jen M., Elenitsas R., Rubin A.I. (2018). Surreptitious apple cider vinegar treatment of a melanocytic nevus: Newly described histologic features. J. Cutan. Pathol..

[14] Johnston C.S., Gaas C.A. (2006). Vinegar: Medicinal uses and antiglycemic effect. MedGenMed.

[15] Nassiri-Asl M., Hosseinzadeh H. (2016). Review of the Pharmacological Effects of *Vitis vinifera* (Grape) and its Bioactive Constituents: An Update. Phyther. Res..

[16] Nishidai S., Nakamura Y., Torikai K., Yamamoto M., Ishihara N., Mori H., Ohigashi H. (2000). Kurosu, a traditional vinegar produced from unpolished rice, suppresses lipid peroxidation in vitro and in mouse skin. Biosci. Biotechnol. Biochem..

[17] Zhang X.L., Zheng Y., Xia M.L., Wu Y.N., Liu X.J., Xie S.K., Wu Y.F., Wang M. (2020). Knowledge domain and emerging trends in vinegar research: A bibliometric review of the literature from WOSCC. Foods.

[18] Gullo M., Verzelloni E., Canonico M. (2014). Aerobic submerged fermentation by acetic acid bacteria for vinegar production: Process and biotechnological aspects. Process Biochem..

[19] Thomas S., Izard J., Walsh E., Batich K., Chongsathidkiet P., Clarke G., Sela D.A., Muller A.J., Mullin J.M., Albert K. (2017). The host microbiome regulates and maintains human health: A primer and perspective for non-microbiologists. Cancer Res..

[20] Croxen M.A., Law R.J., Scholz R., Keeney K.M., Wlodarska M., Finlay B.B. (2013). Recent advances in understanding enteric pathogenic Escherichia coli. Clin. Microbiol. Rev..

[21] DeDent A., Kim H.K., Missiakas D., Schneewind O. (2012). Exploring Staphylococcus aureus pathways to disease for vaccine development. Semin. Immunopathol..

[22] Thomer L., Schneewind O., Missiakas D. (2016). Pathogenesis of Staphylococcus aureus Bloodstream Infections. Annu. Rev. Pathol. Mech. Dis..

[23] Nobile C.J., Johnson A.D. (2015). Candida albicans Biofilms and Human Disease. Annu. Rev. Microbiol..

[24] Carson C.F., Ash O., Chakera A. (2017). In vitro data support the investigation of vinegar as an antimicrobial agent for PD-associated Pseudomonas exit site infections. Nephrology.

[25] Barata A., Malfeito-Ferreira M., Loureiro V. (2012). The microbial ecology of wine grape berries. Int. J. Food Microbiol..

[26] Fleet G.H. (2003). Yeast interactions and wine flavour. Int. J. Food Microbiol..

[27] Brand-Williams W., Cuvelier M.E., Berset C. (1995). Use of a Free Radical Method to Evaluate Antioxidant Activity. LWT Food Sci. Technol..

[28] Pekkarinen S.S., Stöckmann H., Schwarz K., Heinonen I.M., Hopia A.I. (1999). Antioxidant activity and partitioning of phenolic acids in bulk and emulsified methyl linoleate. J. Agric. Food Chem..

[29] Śnieżek E., Szumska M., Janoszka B. (2016). Assessing antioxidative properties of selected plant-based products in terms of their usability as additives to thermally treated high protein food. Food Sci. Technol. Qual..

[30] Singleton V., Rossi J. (1985). Colorimetry of Total Phenolics with Phosphomolybdic-Phosphotungstic Acid Reagents. Am. J. Enol. Viticult..

[31] (2015). CLSI M07-A10 Methods for Dilution Antimicrobial Susceptibility Tests for Bacteria That Grow Aerobically.

[32] (2008). CLSI Reference Method for Broth Dilution Antifungal Susceptibility Testing of Yeasts.

[33] Kwiatkowski P., Mnichowska-Polanowska M., Pruss A., Dzięcioł M., Masiuk H. (2017). Activity of essential oils against Staphylococcus aureus strains isolated from skin lesions in the course of staphylococcal skin infections. Herba Pol..

[34] Ho C.W., Lazim A.M., Fazry S., Zaki U.K.H.H., Lim S.J. (2017). Varieties, production, composition and health benefits of vinegars: A review. Food Chem..

[35] Suárez Valles B., Pando Bedriñana R., Fernández Tascón N., González Garcia A., Rodríguez Madrera R. (2005). Analytical differentiation of cider inoculated with yeast (Saccharomyces cerevisiae) isolated from Asturian (Spain) apple juice. LWT Food Sci. Technol..

[36] Battcock M., Azam-Ali S. (1998). Fermented Frutis and Vegetables. A Global Perspective.

[37] Capozzi V., Fragasso M., Romaniello R., Berbegal C., Russo P., Spano G. (2017). Spontaneous food fermentations and potential risks for human health. Fermentation.

[38] Kántor A., Kačániová M., Kluz M. (2015). Natural microflora of wine grape berries. J. Microbiol. Biotechnol. Food Sci..

[39] Ubeda C., Hidalgo C., Torija M.J., Mas A., Troncoso A.M., Morales M.L. (2011). Evaluation of antioxidant activity and total phenols index in persimmon vinegars produced by different processes. LWT. Food Sci. Technol..

[40] Niculescu V.-C., Paun N., Ionete R.-E., Jordão A.M., Cosme F. (2017). Grapes and Wines—Advances in Production, Processing, Analysis and Valorization.

[41] Aldrete-Tapia J.A., Miranda-Castilleja D.E., Arvizu-Medrano S.M., Hernández-Iturriaga M. (2018). Selection of Yeast Strains for Tequila Fermentation Based on Growth Dynamics in Combined Fructose and Ethanol Media. J. Food Sci..

[42] Bakir S., Devecioglu D., Kayacan S., Toydemir G., Karbancioglu-Guler F., Capanoglu E. (2017). Investigating the antioxidant and antimicrobial activities of different vinegars. Eur. Food Res. Technol..

[43] Sengun I.Y., Kilic G., Ozturk B. (2020). Screening physicochemical, microbiological and bioactive properties of fruit vinegars produced from various raw materials. Food Sci. Biotechnol..

[44] Kadiroğlu P. (2018). FTIR spectroscopy for prediction of quality parameters and antimicrobial activity of commercial vinegars with chemometrics. J. Sci. Food Agric..

[45] Bjornsdottir K., Breidt F., McFeeters R.F. (2006). Protective effects of organic acids oil survival of Escherichia coli O157:H7 in acidic environments. Appl. Environ. Microbiol..

[46] Sanarico D., Motta S., Bertolini L., Antonelli A. (2003). HPLC determination of organic acids in traditional balsamic vinegar of Reggio Emilia. J. Liq. Chromatogr. Relat. Technol..

[47] Mella O., Rose D., Weller C. (2013). Effects of malting and fermentation on the amount of reducing sugars and soluble proteins and free amino acids in white macia and red tannin sorghum flour. Tanzan. Food Nutr. Cent..

[48] Kong Y., Zhang L.L., Sun Y., Zhang Y.Y., Sun B.G., Chen H.T. (2017). Determination of the Free Amino Acid, Organic Acid, and Nucleotide in Commercial Vinegars. J. Food Sci..

[49] Ryssel H., Kloeters O., Germann G., Schafer T., Wiedemann G., Oehlbauer M. (2009). The antimicrobial effect of acetic acid—An alternative to common local antiseptics?. Burns.

[50] Entani E., Asai M., Tsujihata S., Tsukamoto Y., Ohta M. (1998). Antibacterial action of vinegar against food-borne pathogenic bacteria including Escherichia coli O157:H7. J. Food Prot..

[51] Park S.Y., Kang S., Ha S. (2016). Do Antimicrobial effects of vinegar against norovirus and Escherichia coli in the traditional Korean vinegared green laver (Enteromorpha intestinalis) salad during refrigerated storage. Int. J. Food Microbiol..

[52] Pinto T.M.S., Neves A.C.C., Leao M.V.P., Jorge A.O.C. (2008). Vinegar as an antimicrobial agent for control of Candida spp. in complete denture wearers. J. Appl. Oral Sci..

[53] Yagnik D., Serafin V., Shah A.J. (2018). Antimicrobial activity of apple cider vinegar against Escherichia coli, Staphylococcus aureus and Candida albicans; downregulating cytokine and microbial protein expression. Sci. Rep..

[54] Brul S., Coote P. (1999). Preservative agents in foods: Mode of action and microbial resistance mechanisms. Int. J. Food Microbiol..

[55] Fernandes A.C.F., de Souza A.C., Ramos C.L., Pereira A.A., Schwan R.F., Dias D.R. (2019). Sensorial, antioxidant and antimicrobial evaluation of vinegars from surpluses of physalis (Physalis pubescens L.) and red pitahaya (Hylocereus monacanthus). J. Sci. Food Agric..

[56] Mietton-Peuchota M., Milisica V., Noilet P. (2002). Grape must concentration by using reverse osmosis. Comparison with chaptalization. Desalination.

[57] Oh D.H., Marshall D.L. (1993). Antimicrobial activity of ethanol, glycerol monolaurate or lactic acid against Listeria monocytogenes. Int. J. Food Microbiol..

[58] Liu Q., Tang G.-Y., Zhao C.-N., Gan R.-Y., Li H.-B. (2019). Antioxidant Activities, Phenolic Profiles, and Organic Acid Contents of Fruit Vinegars. Antioxidants.

[59] Giovinazzo G., Grieco F. (2015). Functional Properties of Grape and Wine Polyphenols. Plant Foods Hum. Nutr..

[60] Chong J., Poutaraud A., Hugueney P. (2009). Metabolism and roles of stilbenes in plants. Plant Sci..

[61] Spáčil Z., Nováková L., Solich P. (2008). Analysis of phenolic compounds by high performance liquid chromatography and ultra performance liquid chromatography. Talanta.

[62] Cantos E., Espín J.C., Tomás-Barberán F.A. (2002). Varietal differences among the polyphenol profiles of seven table grape cultivars studied by LC-DAD-MS-MS. J. Agric. Food Chem..

[63] Castillo-Muñoz N., Gómez-Alonso S., García-Romero E., Hermosín-Gutiérrez I. (2007). Flavonol profiles of Vitis vinifera red grapes and their single-cultivar wines. J. Agric. Food Chem..

[64] Mattivi F., Zulian C., Nicolini G., Valenti L. (2002). Wine, biodiversity, technology, and antioxidants. Ann. N. Y. Acad. Sci..

[65] Bagchi D., Bagchi M., Stohs S.J., Das D.K., Ray S.D., Kuszynski C.A., Joshi S.S., Pruess H.G. (2000). Free radicals and grape seed proanthocyanidin extract: Importance in human health and disease prevention. Toxicology.

[66] Giusti F., Caprioli G., Ricciutelli M., Vittori S., Sagratini G. (2017). Determination of fourteen polyphenols in pulses by high performance liquid chromatography-diode array detection (HPLC-DAD) and correlation study with antioxidant activity and colour. Food Chem..

[67] Brighenti E., Casagrande K., Cardoso P.Z., da Silveira Pasa M., Ciotta M.N., Brighenti A.F. (2017). Total polyphenols contents in different grapevine varieties in highlands of southern brazil. BIO Web Conf..

[68] Bin D., He B., Shi P., Li F., Li J., Zhu F. (2012). Phenolic content and antioxidant activity of wine grapes and table grapes. J. Med. Plants Res..

[69] Lu Y., Liu Y., Lv J., Ma Y., Guan X. (2020). Changes in the physicochemical components, polyphenol profile, and flavor of persimmon wine during spontaneous and inoculated fermentation. Food Sci. Nutr..

[70] König H., Claus H. (2018). A Future Place for Saccharomyces Mixtures and Hybrids in Wine Making. Fermentation.

[71] Palla M., Blandino M., Grassi A., Giordano D., Sgherri C., Frank Quartacci M., Reyneri A., Agnolucci M., Giovannetti M. (2020). Characterization and selection of functional yeast strains during sourdough fermentation of different cereal wholegrain flours. Sci. Rep..

[72] Minnaar P., Jolly N., Beukes L., Benito S. (2021). Effect of alcoholic and acetous fermentations on the phenolic acids of Kei-apple (Dovyalis caffra L.) fruit. J. Sci. Food Agric..

[73] Samoticha J., Wojdyło A., Chmielewska J., Nofer J. (2019). Effect of Different Yeast Strains and Temperature of Fermentation on Basic Enological Parameters, Polyphenols and Volatile Compounds of Aurore White Wine. Foods.

[74] Mareček V., Mikyška A., Hampel D., Čejka P., Neuwirthová J., Malachová A., Cerkal R. (2017). ABTS and DPPH methods as a tool for studying antioxidant capacity of spring barley and malt. J. Cereal Sci..

[75] Noreen H., Semmar N., Farman M., McCullagh J. (2017). Measurement of total phenolic content and antioxidant activity of aerial parts of medicinal plant Coronopus didymus. Asian Pac. J. Trop. Med..

[76] Alberto M.R., Farías M.E., Manca De Nadra M.G. (2001). Effect of gallic acid and catechin on Lactobacillus hilgardii 5w growth and metabolism of organic compounds. J. Agric. Food Chem..

[77] Dávalos A., Bartolomé B., Gómez-Cordovés C. (2005). Antioxidant properties of commercial grape juices and vinegars. Food Chem..

[78] Vaquero M.J.R., Manca De Nadra M.C. (2008). Growth parameter and viability modifications of escherichia coli by phenolic compounds and argentine wine extracts. Appl. Biochem. Biotechnol..

[79] Papadopoulou C., Soulti K., Roussis I.G. (2005). Potential Antimicrobial Activity of Red and White Wine Phenolic Extracts against Strains of Staphylococcus aureus, Escherichia coli and Candida albicans. Food Technol. Biotechnol..

[80] Rotava R., Zanella I., da Silva L.P., Manfron M.P., Ceron C.S., Alves S.H., Krakow A.K., Santos J.P.A. (2009). Antibacterial, antioxidant and tanning activity of grape by-product. Cienc. Rural.

[81] Thimothe J., Bonsi I.A., Padilla-Zakour O.I., Koo H. (2007). Chemical characterization of red wine grape (Vitis vinifera and Vitis interspecific hybrids) and pomace phenolic extracts and their biological activity against Streptococcus mutans. J. Agric. Food Chem..

